# Multistakeholder perspectives on geographical accessibility to emergency obstetric care in Benin City, Nigeria

**DOI:** 10.1016/j.afjem.2026.100963

**Published:** 2026-03-13

**Authors:** Uchenna Gwacham-Anisiobi, Funmi Adio, Alero Ogbebor, Michael Ezeanochie, Aduragbemi Banke-Thomas

**Affiliations:** a Centre for Clinical Trials, Research, and Implementation Science, College of Medicine, University of Lagos, Lagos State, Nigeria; b Department of Community Health, University of Benin Teaching Hospital, Benin City, Edo State, Nigeria; c Department of Obstetrics and Gynaecology, University of Benin Teaching Hospital, Benin City, Edo State, Nigeria; d Faculty of Epidemiology and Population Health, London School of Hygiene and Tropical Medicine, UK

**Keywords:** Emergency obstetric care, Maternal health, Urban health systems, Nigeria, Access to care

## Abstract

**Introduction:**

In many Nigerian cities, travel to emergency obstetric care (EmOC) remains challenging, and the so-called urban advantage is shrinking. Benin City, Nigeria, has four major referral hospitals providing EmOC, yet maternal mortality remains very high. While facility-based deliveries are common, many women still face significant delays in reaching timely, appropriate care. This study explored women’s and stakeholders’ perspectives on EmOC geographical accessibility in this rapidly urbanising city.

**Methods:**

This descriptive qualitative study was conducted in four referral hospitals in Benin City, Nigeria. In-depth interviews were conducted with 44 purposively recruited women who had experienced obstetric emergencies, alongside 11 key stakeholders, including health service planners and policymakers. Women were recruited from hospital settings and communities in three local government areas identified as having the poorest geographical access to EmOC. Thematic analysis followed Braun and Clarke’s six-step approach.

**Results:**

Four themes emerged from our study: 1) travel challenges force some women to use unsafe transport and seek informal care in emergencies, 2) bypassing non-preferred facilities prolonged travel to obstetric care, 3) systemic inefficiencies further complicates EmOC geographical access, and 4) multi-sectoral action needed to improve EmOC geographical access. Women described unsafe roads, lack of transport, and security concerns, particularly at night, leading to delays or resorting to traditional birth attendants. Referral inefficiencies, workforce shortages, and inadequate facility readiness compounded these delays. Participants proposed infrastructure upgrades, birth preparedness, improved insurance coverage, and stronger referral coordination to reduce in-transit delays and ensure equitable access.

**Discussion:**

Timely access to EmOC in urban settings is undermined by the intersection of spatial inequities, system dysfunction, and unreliable service availability. Addressing these challenges requires integrated infrastructure planning, strengthened referral coordination, and investment in health workforce retention. Without effective implementation of existing policies and targeted support for high-burden areas, maternal health inequities will persist even in urban contexts.

## African relevance

• Geographic barriers in urban African settings delay timely access to emergency obstetric care, especially for women in peripheral and underserved communities.
• Issues with geographical accessibility to emergency obstetric care affect travel choices of pregnant women to care including forcing them to seek locally available informal care.
• Fragmented referral systems, limited ambulance availability, and unreliable staffing exacerbate delays in travel to reach care.
• Coordinated investments in roads, emergency transport, and referral system governance are crucial to improving geographical access to care in African cities.

## Introduction

Maternal mortality and stillbirths have declined globally in recent decades, yet Africa continues to bear a disproportionate burden. In 2023, Nigeria, Africa’s most populous country, accounted for an estimated 29 % (75,000) of global maternal deaths and 10 % (184,000) of global stillbirths [[1], [2], [3]]. Timely access to emergency obstetric care (EmOC) provided by skilled health personnel (SHP), such as doctors and nurses, at health facilities has been shown to reduce as much as 50 % of maternal deaths and 75 % of stillbirths [4]. As per global guidelines, EmOC is a service package comprising nine evidence-based interventions, otherwise known as signal functions, including seven basic (injectable antibiotics, oxytocics, anticonvulsants, manual removal of placenta, removal of retained products, assisted vaginal delivery and neonatal resuscitation) and two comprehensive functions (blood transfusion and caesarean section) [5].

Delays in accessing EmOC are often retrospectively understood using the ‘three delays’ model: delay in deciding to seek care (Delay 1), delay in geographically accessing or reaching an appropriate health facility (Delay 2), and delay in receiving adequate care upon arrival (Delay 3) which together contribute to preventable maternal and perinatal deaths [6]. While all delays increase risk of poor pregnancy outcomes, studies have shown that at least a third of maternal deaths are attributable to delays in reaching an appropriate EmOC facility (Delay 2), which are usually due to issues relating to transport, travel time, terrain, weather, and physical barriers [7,8]. Further, evidence shows that maternal deaths are more likely when referral journeys exceed 30 min, and stillbirth risk increases with travel times of over 10 min [9,10]. Urbanisation further complicates challenges relating to delay in reaching care [11].

With Nigeria’s urban population projected to exceed 70 % by 2050 [12,13], health systems in cities face additional pressure. Expansion of slums, traffic congestion, poor road conditions, and extreme weather further impair access to EmOC in cities. Based on a 2024 analysis of EmOC geographical accessibility in Nigeria, Benin City has some of the longest EmOC travel times among Nigerian cities over one million in population, with marked intra-urban inequities [14]. Published studies that sought to explore issues relating to EmOC geographical accessibility in similar low-resource cities have mostly focused on women’s perspectives [15,16], while studies using the three delays framework often lack contextual detail on the second delay in Nigeria [17]. Understanding the perspectives of both service users; women and their families who directly experience delays in reaching EmOC and stakeholders responsible for service planning and delivery is essential for shaping effective strategies to advance universal health coverage. The aim of this study is to explore perspectives of stakeholders on EmOC geographical accessibility in Benin City.

## Methods

### Study design

We adopted a descriptive qualitative design to explore diverse stakeholder perspectives on EmOC geographical access in Benin City. This design was selected to capture rich, context-specific accounts, while generating insights directly relevant for comprehensive health system response. We followed the Consolidated Criteria for Reporting Qualitative Research in writing up the study [18].

### Study setting

This study was conducted in Benin City, the capital of Edo State, Nigeria, with a population of approximately 1.9 million, of whom just over a quarter are women of reproductive age [19]. The city hosts public, private, and faith-based health facilities offering varying levels of maternal healthcare [20], reflecting Nigeria’s three-tiered health system: primary, secondary, and tertiary level care [21]. Comprehensive EmOC is provided at secondary and tertiary levels, which are typically hospitals, while primary facilities, which are typically clinics and primary health care centres offer basic EmOC and refer complicated cases upward. Women may self-refer themselves or may be referred by a health worker to such facilities providing comprehensive EmOC [22].

The city has four major referral hospitals that offer routine obstetric care and manage referred emergency obstetric cases, which account for approximately 20 % of their annual deliveries [23]. These include the University of Benin Teaching Hospital (federal tertiary, ∼2500 annual deliveries), Edo Specialist Hospital (state-run tertiary, ∼2000 annual deliveries), Central Hospital Benin City (state-run secondary, ∼2000 annual deliveries), and St. Philomena Catholic Hospital (missionary secondary, ∼1500 annual deliveries). Despite the relatively high rate of facility-based deliveries compared to other Nigerian cities [24,25], institutional maternal mortality ratios reported in the selected facilities since year 2014 have ranged from 395 to as high as 2292 per 100,000 live births [[26], [27], [28], [29]].

### Recruitment and sampling of study participants

Study participants included women aged 18 years and above who had experienced obstetric emergencies, community women, heads of obstetrics and gynaecology departments of the city’s four largest referral hospitals, policymaker and service planners from the state’s parastatals including the Edo State Emergency Medical Services (EdoEMS). Stakeholders were purposefully selected based on their roles in service planning, policy, access, provision or receipt of EmOC.

Within stakeholder groups, participants were purposively sampled to ensure diverse characteristics relevant to understand the topic were represented. For example, policymakers and service planners from agencies managing the different levels of service provision (primary, secondary, and tertiary) and women based on their marital status, education, employment status, and obstetric history.

Women were recruited from hospitals and communities. In hospitals, women were recruited after confirmation of them presenting in obstetric emergencies, receiving EmOC and following clinical stabilisation. Community women were identified via community health workers and snowball sampling in areas in the city established from previous research to have limited EmOC geographical access [14]. Other stakeholder groups were recruited via letters sent to their offices. All recruitment was done by the research team with their availability confirmed and informed consent obtained a priori.

### Data collection

In-depth interviews were conducted with women in private rooms within the hospital or quiet community locations, while stakeholder interviews took place in their offices, using a piloted guide informed by previous research, covering decision-making, travel, delays, and coping mechanisms [16]. While the content and domains of the original guide were retained, minor adjustments were made to accommodate the local context in Benin City. Interviews were conducted in English or Pidgin English, depending on participant preference. All interviews were conducted directly by one of three female clinician-researchers on the research team who were trained in qualitative methods and fluent in both languages, eliminating the need for external interpreters and minimising translation bias. Interviews explored decision-making, travel, delays, and coping mechanisms for women, and understanding of EmOC access, system barriers, and ongoing or proposed interventions for stakeholders.

Interviews lasted 30–45 min, were audio-recorded with participant consent [30]. Reflective field notes were taken throughout. Data collection occurred from May to August 2024 when thematic saturation (i.e. no new theme emerging) was attained. All study data, including audio recordings, transcripts, and field notes, were stored securely on password-protected computers accessible only to the research team. Physical documents were kept in locked cabinets at the research office. Audio files were encrypted during transfer, and transcripts de-identified to maintain confidentiality. Data will be retained securely for five years from 2024 in line with UBTH ethics committee requirement.

### Data analysis

Braun and Clarke’s six-steps which are becoming familiar with the data, generating initial codes, searching for themes, reviewing themes, defining and naming themes, and producing the report guided our analysis [31]. Audio-recordings were transcribed verbatim. Using an inductive approach, initial codes were generated by UGA and FA without forcing data into pre-defined categories [32]. Related codes were grouped into categories and refined into themes through team discussions with the research team and iterative review. This was done to mitigate potential biases and ensure that participants’ experiences were centred in the analysis [30,31]. Member checking with the interviewee allowing them to confirm or correct the themes was conducted [30]. Analysis was supported with NVivo 14 (QSR International, Memphis, USA).

### Ethical considerations

Ethical approval was obtained from UBTH Health Research and Ethics Committee (ADM/E 22/A/VOL. VII/14830112952). All participants provided written informed consent. They were informed about the study purpose, procedures, voluntary participation, and their right to withdraw at any time. Confidentiality and anonymity were ensured throughout the study.

## Results

There were 55 participants in this study, including 44 women (34 from hospitals and 10 from communities). The remaining 11 participants were key informants, divided into three main categories: one policy maker, five service planners representing the State Health Ministry, the State Health Insurance Commission, the Primary Health Care Agency, and Emergency Medical Services; and five clinical leaders/obstetricians representing four different hospitals [Table 1, Table 2].

**Table 1:** Sociodemographic characteristics of women included in the study.

**Characteristic**	**Number**	**Percentage**
**Age (years)**		
18–24	8	18
25–34	25	57
35–44	10	23
≥45	1	2
**Educational attainment**		
No formal education	5	11
Primary	9	20
Secondary	21	48
Tertiary	9	20
**Occupational status**		
Employed	35	80
Unemployed	9	20
**Marital status**		
Married	39	89
Single	5	11
**Parity**		
Nulliparous	5	11
Primiparous	13	30
Multiparous	26	59

**Table 2:** Distribution of key informant participants by role category and affiliation.

**Category**	**Number of participants**	**Range of affiliation**
Policymaker	1	State Ministry of Health
Service planners	5	State Ministry of Health, State Health Insurance Commission, Primary Health Care Agency, Emergency Medical Services.
Heads of Obstetric Departments/ Obstetricians	5	Four unique Hospitals (coded 1–4)

Four key themes emerged (Table 3). These were: 1) Travel challenges force some women to use unsafe transport and seek informal care in emergency, 2) Bypassing non-preferred facilities prolonged travel to obstetric care, 3) Systemic inefficiencies further complicates EmOC geographical access, and 4) Multi-sectoral action needed to improve EmOC geographical access. Detailed thematic framework shown in Supplementary Table 1.

**Table 3:** Summary of themes and subthemes.

**Theme**	**Subthemes**
1. Travel challenges force some women to use unsafe transport and seek informal care in emergency	1.1 Physical and environmental barriers 1.2 Transport availability and modality 1.3 Safety and security concerns 1.4 Resort to informal care
2. Bypassing non-preferred facilities prolonged travel to obstetric care	2.1 Perceived quality and trust decisions 2.2 Social networks shaping bypass 2.3 Financial trade-offs and selective referrals 2.4 Longer travel and delayed arrival
3. Systemic inefficiencies further complicate emergency obstetric care geographical access	3.1 Referral system breakdowns 3.2 Emergency transport and equipment gaps 3.3 Human resource shortages 3.4 Facility-level referral delays
4. Multi-sectoral action needed to improve emergency obstetric care geographical access	4.1 Infrastructure and urban planning solutions 4.2 Emergency transport and referral coordination 4.3 Workforce retention and incentives 4.4 Financial protection and birth preparedness

### Travel challenges force some women to use unsafe transport and seek informal care in emergency

Women in peripheral and densely populated informal settlements described how poor roads, flooding, and the absence of formal transport options made travel during obstetric emergencies extremely difficult. This challenge was not lost on policymakers, as many of them recognised the poor road infrastructure, which is worsened by inclement weather. Several women noted that the difficulty of accessing EmOC significantly influenced their decisions. Indeed, some women reported that they were left with little choice but to seek informal care, even during obstetric emergencies.

“*The road is bad and there is no hospital at all in that environment. It’s only school… but there is land to build a hospital*” (Multiparous woman 18, community).

“*Those Evbuotubu, Sapele Road, even Aduwawa axis, when it rains you can't pass that place. So, if a woman is in labour or has emergency, she can't pass through there*” (Service planner 1).

*“On my own, I don’t like TBA [traditional birth attendant], but I go there because there is no hospital nearby. I was in so much pain [while in labour] and bleeding. If there was a hospital nearby, I would have gone!'* (Multiparous woman 9, community)."

Motorcycles were often the only feasible means of transport, despite safety concerns and the physical discomfort of navigating long, rough roads while in labour, especially when referral to tertiary facilities was needed.

*“The main mode of transportation here is okada [motorbike] because the roads are very bad. It is very difficult to find taxi especially at night,… unless you have a neighbour that has a car and can take you to the hospital”* (Multiparous woman 24, hospital).

### Bypassing non-preferred facilities prolonged travel to obstetric care

Although proximity was important, women often bypassed nearby facilities in favour of hospitals they perceived to offer better quality of care. This decision was often informed by personal experiences, prior loss, or accounts within their social networks. Such choices were not merely about physical access but were rooted in trust, emotional reassurance, and the fear of complications. One woman, who had previously lost a baby, explained that:

“…*there were other facilities around me, but I have a church member who has given birth here, and my husband and my pastor also asked me to come here and register… I didn’t want what happened to my last baby that died to happen again*” (Multiparous woman 4, hospital).

However, other stakeholders highlighted that bypassing had consequences. Longer travel often meant arriving later in labour or during emergencies. Policy and service planners acknowledged additional financial trade-offs women made during emergencies. Clinical leaders reported that women sometimes declined formal referrals, opting instead for facilities that were less costly but ill-equipped or farther away, further delaying their access to definitive care.

“*We know that some pregnant women face financial challenges… they will tell you that those places are expensive and that they cannot afford it, even at the expense of their lives*,” (Obstetric Department Lead, Hospital 4).

### Systemic inefficiencies further complicate EmOC geographical access

Stakeholders highlighted widespread health system failures that delayed geographical access. Women reported issues relating to unavailability of service in facilities local to them resulting in them needing to travel farther for care in an emergency and in some cases needing to travel to multiple facilities before definitive care can be accessed. Reasons for service unavailability included maternity staff unavailability due to them being on leave, with no one assigned to cover their absence, equipment and ancillary service not being provided, and facility being locked.

“*We were told that she was given three months' maternity leave, and there was no replacement for her…*” (Multiparous woman 26, community).

“*I trekked from my house to a hospital for about 15min, but I couldn’t get care there because the hospital was locked… I then walked for another 30min to another hospital… but was told there was no lab equipment at 11pm. Then I walked to this hospital and arrived at about past 12am*” (Primiparous woman 13, hospital).

The policymaker and service planners agreed that the number of SHP are not sufficient but pointed to the ongoing brain drain and non-replacement of departing personnel as the main driver for this. Service planners identified workforce shortages as a serious challenge with many facilities being understaffed, particularly in suburban areas.

“*These days that we have brain drain syndrome… We obviously don’t have enough… Is the government employing? No*” (Obstetric Department Lead, Hospital 4).

Several women described being referred between facilities without coordination and sometimes with significant waits for transport. In response, service planners highlighted that facilities frequently lacked ambulances, requiring women to organise their own transport, sometimes even in life-threatening situations. Where ambulances exist, urban traffic congestion was cited as a significant contributor to delays, particularly when combined with distance and poor coordination. They also flagged that another problem was with delayed referrals which they said occurred because providers from some lower-level facilities tried to manage complex cases beyond their capacity. These systemic inefficiencies were seen to be introducing multiple failure points in the care continuum, thereby affecting geographical accessibility and outcomes of care.

“*I stayed like an hour [at the first facility]… I had to wait for a cab to pick me… it took like two hours before getting to [the hospital]*” (Multiparous woman 13, hospital).

“*There is no ambulance that will convey them immediately… There could be a lot of traffic [congestion] and then there is no means of boycotting that particular road*,” (Obstetric Department Lead, Hospital 4).

“*Facilities that are not competent enough keep patients till it is almost too late. By the time of referral, little or no intervention can be done*” (Obstetric Department Lead, Hospital 3).

### Multi-sectoral action needed to improve EmOC geographical access

Stakeholders across all groups advocated for cross-sectoral solutions to address barriers. Women advocated for new or upgraded facilities in underserved areas. For the existing facilities, especially those with difficult accessibility, service planners emphasised the need to retain staff in these areas through training and incentives, as this would reduce the distance women need to travel to access EmOC.

“*Since they said they do not have enough facility there to take care of my health, so I believe [the government] should do something about that so they would stop needing to refer*” (Nulliparous woman 5, community).

“*We need to employ personnel, train and retrain them. Give them incentives, in order to stay [in the suburbs]*,” (Obstetric Department Lead, Hospital 4).

Improving emergency transport and referral systems was another priority across stakeholder groups. Many women expressed a desire for facilities to be equipped with ambulances that could ease the burden of long and unpredictable journeys, particularly by navigating traffic. The policymaker reported that some promising initiatives were already underway. However, awareness remained low, so even where emergency transport services existed, many women still had to arrange their own travel, leading to avoidable delays. Even when transport was available in facilities, poor referral coordination forces women into unnecessary or repeated journeys. Service planners therefore called for better referral coordination to minimise unnecessary transfers and repeated journeys between facilities.

“*I wish they have an emergency vehicle… with this sound [siren], so that if there is any hold-up on the way it can help*” (Primiparous woman 7, hospital).

“*We have started with emergency medical transport… standby ambulances… and we are doing the three pilot LGAs for now*,” (Service planner 1).

“*Most women don’t know about it and the availability of ambulance services*,” (Service planner 5).

“*There should be a policy… once a referral is made, there should be a means of communicating with the receiving facility even before the patient leaves*” (Obstetric Department Lead, Hospital 4).

In addition to response at point-of-need strategies above, the policymaker proposed preventive strategies, including antenatal birth preparedness education and encouraging planned relocation near due dates, to reduce the risk of long or urgent travel when labour begins.

“*Health workers should teach [women] about birth preparedness and complication readiness*” (Policymaker 1).

## Discussion

This study provides critical insights into how women and key stakeholders in Benin City experience and understand EmOC geographical accessibility. While cities are often assumed to offer improved maternal health outcomes due to service concentration [33,34], our findings show that urban living does not necessarily equate to accessibility. Women residing in suburban and slum areas experience significant geographic and infrastructural barriers that delay timely care and, in some cases, lead them to seek services from informal providers. Similar patterns of intra-urban inequity have been documented in Lagos, Dakar and other rapidly urbanising African cities, where proximity to facilities does not translate into timely access due to congestion, uneven infrastructure, and spatial marginalisation [16,35]. Our findings reinforce growing evidence that the so-called urban advantage is often undermined by spatial injustice and system unreliability.

Accounts of women navigating unsafe, impassable roads, especially during the rainy season, highlight how environmental and infrastructural neglect deepen maternal geo-access vulnerability. Many participants described the near impossibility of travelling at night, citing the absence of transport, security concerns, and risks associated with relying on motorcycles. Comparable infrastructural and environmental barriers have been reported across sub-Saharan Africa, where poor road conditions, flooding and insecurity prolong emergency travel even in urban settings [16,36]. Crucially, some women, while recognising the risks, opted for traditional birth attendants due to lack of formal services nearby. This recourse to informal care in emergency, when survival depends on rapid access to SHP, illustrates the dilemma women sometimes find themselves in emergency.

The issue of bypassing nearer facilities in favour of more distant ones perceived to offer better quality care emerged strongly in our study. While bypassing has been documented elsewhere [16,37,38], our findings deepen understanding of its local drivers: past negative experiences, cost-related rejections of referrals, and advice from trusted community members. The willingness to travel longer distances in emergencies highlights a troubling trade-off: proximity versus perceived competence. Consistent with studies from Lagos, Dakar and other African cities, women in our study were willing to incur longer travel times to access facilities perceived as more competent, demonstrating that perceived quality and trust can override geographical proximity in emergency decision-making [16,36,39]. These patterns highlight the importance of ensuring consistent quality across all facilities, not only to reduce physical delays but to rebuild trust in nearby services.

Stakeholders acknowledged systemic inefficiencies, including the lack of ambulances, poor inter-facility communication, and closures or understaffing during critical hours. Narratives of women being moved between facilities without coordination or assurance of service readiness reflects broader structural gaps. Some facilities keep patients too long before making referrals, effectively narrowing the time window for life-saving interventions. One woman’s experience of trekking between multiple facilities late at night, while in distress, and repeatedly denied care due to lack of resources, captures the cumulative toll of system dysfunction. These issues have been similarly reported in prior research from other urban African settings [[39], [40], [41], [42]]. What emerges is not merely a problem of infrastructure, but of unreliability where even geographically proximate facilities may be non-functional, ill-equipped, or unstaffed when urgently needed [37,43]. Such system-level dysfunction has been shown across SSA to be a major contributor to preventable maternal and perinatal mortality [10,26].

The policy implications of this study point to the need for coordinated, multisectoral action that addresses both spatial and systemic drivers affecting EmOC geographical accessibility. Infrastructure investments particularly in roads, emergency transport, and primary and secondary facility upgrades especially in underserved city areas, should be prioritised. The call for spatial justice in health planning is particularly pressing considering the rapid expansion of Nigeria’s urban peripheries, where investments in infrastructure must be matched by the equitable deployment of SHP [44,45]. To reduce referral-related delays, coordination should leverage digital platforms, mobile networks, and expert call centres, drawing lessons from successful models like Ghana’s obstetric referral system [46]. In Benin City, pilot initiatives such as the emergency transport system implemented with Emergency Response Africa [47] were reported to be underused, largely due to limited awareness among women and communities about the service. This underscores the need not only for infrastructure and systems, but also for community sensitisation so that available emergency resources are effectively utilised. Similar approaches have been successful in other contexts; for instance, integrating digital platforms and mobile networks in urban slums of Dhaka, Bangladesh, improved EmOC access by enhancing referral coordination and communication [48].

The multistakeholder lens used in this study is a strength as it illustrates a tension between community expectations, government capacity, and the design of emergency interventions. Recognising these misalignments is crucial for designing feasible, context-specific interventions that bridge the gap between women’s needs and system capabilities. However, this study has some limitations which must be acknowledged. It was conducted in a single urban setting, which may restrict transferability, although qualitative research seeks depth of insight rather than generalisability. Recruitment through facilities and gatekeepers may have under-represented women with the most adverse outcomes, a limitation noted in similar studies [49]. However, gathering insights from multiple stakeholders provides a comprehensive understanding of EmOC access, enhancing the credibility and practical relevance of the findings [50].

## Conclusion

Our study findings show that delays in reaching EmOC in Benin City arise from the intersection of multiple issues relating to geography, service reliability, referral breakdowns, and infrastructural neglect. Each issue compounds another, creating a cascade of disadvantage that disproportionately affects women in poorer, peripheral communities. Addressing these issues demands a coordinated response that integrates spatial justice into urban planning, strengthens referral system governance, and ensures that all women, regardless of where they live, can access timely EmOC.

## Credit author statement

UGA: Formal analysis, methodology, writing – original draft, writing – review and editing, FA: conceptualization, data curation, formal analysis, writing – review and editing, AO: Data curation, writing – review and editing, ME: conceptualization, methodology, writing – review and editing, ABT: Conceptualization, formal analysis, methodology, funding, supervision, writing – original draft, writing – review and editing.

## Dissemination of results

Findings from this research were shared with policymakers and key stakeholders during a dissemination meeting held in Edo State. The meeting provided an opportunity to present the results, discuss implications, and gather feedback from those involved in maternal health governance and service delivery. Additionally, the findings will be integrated into ongoing policy discussions to inform improvements in maternal health services in the region.

## Declaration of interest statement

The authors declare that they have no known competing financial interests or personal relationships that could have appeared to influence the work reported in this paper.

## Appendix Supplementary materials

Image, application 1
