# Supplementary material for: Multistakeholder perspectives on geographical accessibility to emergency obstetric care in Benin City, Nigeria
Source: Afr J Emerg Med. 2026 Mar 13;16(2):100963. doi: 10.1016/j.afjem.2026.100963 (PMC12996644; doi:10.1016/j.afjem.2026.100963)
Supplement: Supplementary file 1 [file mmc1.docx]

**Supplementary Table 1**. Thematic framework from multistakeholder perspectives

| **Theme** | **Subtheme** | **Categories** | **Example codes** | **Exemplar quotes** |
| --- | --- | --- | --- | --- |
| Travel challenges force some women to use unsafe transport and seek informal care in emergency | 1.1 Physical & environmental barriers | State of roads, flooding | Bad roads, impassable after rain | “Those Evbuotubu, Sapele Road, even Aduwawa axis, when it rains you can't pass that place.” |
|  | 1.2 Transport availability & modality | Lack of taxis at night; reliance on motorcycles (Okada); no public emergency transport | Difficulty in getting transport, night emergency worse | “The main mode of transportation here is Okada [motorbike] because the roads are very bad. It is very difficult to find taxi especially at night…” |
|  | 1.3 Safety & security concerns | Fear of night travel; security risk influences decisions | Unsafe travel at night’ perception of safety | “Okay, the labour started at 3:30am, we waited till dawn, before going to the hospital.’ ……..’Yes, we waited. You know our community is not very safe. So we do not run into trouble.” |
|  | 1.4 Consequence: resort to informal care | Reason for TBA use | No hospital around, don’t like TBA | “On my own, I don’t like TBA, but I go there because there is no hospital nearby. I was in so much pain [while in labour] and bleeding.” |
| Bypassing non-preferred facilities prolonged travel to obstetric care | 2.1 Perceived quality & trust decisions | Preference for hospitals perceived as better; prior loss shapes choice | Bypass because of perceived quality of care, bypass driven by past loss experience | “There were other facilities around me, but … my husband and my pastor also asked me to come here … I didn’t want what happened to my last baby that did not survive to happen again…” |
|  | 2.2 Social networks & advice shaping bypass | Advice from church members, family, community shapes where women go in emergencies | Family against CS, preference for church intervention | “…I told my husband, he was against it that, C.S again and me I had the faith that let me still try. … So, my mum was like okay, let us go to church. Let us go and try. But before then I do attend one women fellowship where we pray and they still give deliver there. So, I was like okay, let me go there.” |
|  | 2.3 Financial trade-offs & selective referrals | Perceived cost vs. competence trade-off; declining referrals for cost reasons | Cost incurred for emergency care,  Decline referrals | “You also know that some of these pregnant women, they also have financial challenges too. For example, the patient comes and then you want to refer to [Hospital X] or some other places, they will tell those places are expensive, they cannot be able to afford it, at the expense of their life. ” |
|  | 2.4 Consequence: longer travel & delayed arrival | Increased travel time; arrive later in labour/emergency | Going to other facilities increases travel time | “Because they feel don’t have the money to be able to go to where you are referring them to, they go to other places [facilities], and when they get there, the health workers will try, and they will eventually refer them to the very first place that you initially referred them to, and by the time they get there, some complications must have set in” |
| Systemic inefficiencies further complicate EmOC geographical access | 3.1 Referral system breakdowns | Poor coordination; no pre-notification; patients moved between facilities | No referral coordination, delays in transfer | “my younger sister had a surgery, a month later, she started bleeding, as in too much bleeding, they rushed her to the hospital, they did not even touch her, they just referred to X hospital. When they got to X hospital, they said no space. This was around 2am or 3am, no space, nobody was ready to even listen to anybody. We had to leave there again to now private hospital.” |
|  | 3.2 Emergency transport & equipment gaps | Lack of ambulances; lack of facility readiness; blood bank gaps | No ambulance, no blood supply, facility not ready | “The major challenge they face, is the means of getting there early. Some of, sometimes, the, they may have to source for their, for the transportation by themselves at that critical state and then they will go around and then be looking for a vehicle because there is no ambulance that will convey them immediately. |
|  | 3.3 Human resources shortages & hours gaps | Human resource shortages, key staff on leave with no cover | staff shortage, brain drain, no cover for midwife on maternity leave, limited night staff | “The main Nurse at the PHC in our community newly had a baby. We were told that she was given three months' maternity leave, and there was no replacement for her. |
|  | 3.4 Facility-level behaviours that delay referral | Facilities attempt to manage beyond capacity; keep patients too long | Late referral, health workers manage case beyond capacity | “In my experience, health facilities that are not competent enough in managing these patients, keep patients till it is almost too late. By the time of referral, little or no intervention can be carried out. So the important thing is for a facility to know when to refer to another for adequate medical intervention.” |
| Multi-sectoral action needed to improve EmOC geographical access | 4.1 Infrastructure & urban planning solutions | Upgrade roads, building facilities in underserved areas to reduce the need to travel | Build new or repair bad roads, | “It is only the school we have, but there is land to build a hospital. It is there.” |
|  | 4.2 Emergency transport & referral coordination interventions | Ambulance services ( like EdoEMS); hospital pre-notification | Make ambulance available, inform the receiving hospital | “We have started with emergency medical transport. We have standby ambulances, and we are doing this in three pilot LGAs for now” |
|  | 4.3 Workforce & incentives | Retain staff in peripheries; training & incentive packages | Employ more staff, train staff, incentive to stay in underserved rural areas | “We need to employ personnel, train and retrain them. Give them incentives, in order to stay [in the suburbs].” |
|  | 4.4 Financial protection & birth preparedness | Insurance schemes, lower bills, antenatal birth-preparedness education | Antenatal education, insurance coverage | “Healthcare workers should learn to interact more with the women during antenatal clinics and teach them on birth preparedness and complication readiness” |
